# Active Inference, epistemic value, and vicarious trial and error

**DOI:** 10.1101/lm.041780.116

**Published:** 2016-07

**Authors:** Giovanni Pezzulo, Emilio Cartoni, Francesco Rigoli, Léo Pio-Lopez, Karl Friston

**Affiliations:** 1 Institute of Cognitive Sciences and Technologies, National Research Council, 00185 Rome, Italy; 2 The Wellcome Trust Centre for Neuroimaging, UCL, London WC1N 3BG, United Kingdom; 3 Pascal Institute, Clermont University, 63000 Clermont-Ferrand, France; 4 La Sapienza University of Rome, Rome, 00185 Italy

## Abstract

Balancing habitual and deliberate forms of choice entails a comparison of their respective merits—the former being faster but inflexible, and the latter slower but more versatile. Here, we show that arbitration between these two forms of control can be derived from first principles within an Active Inference scheme. We illustrate our arguments with simulations that reproduce rodent spatial decisions in T-mazes. In this context, deliberation has been associated with vicarious trial and error (VTE) behavior (i.e., the fact that rodents sometimes stop at decision points as if deliberating between choice alternatives), whose neurophysiological correlates are “forward sweeps” of hippocampal place cells in the arms of the maze under consideration. Crucially, forward sweeps arise early in learning and disappear shortly after, marking a transition from deliberative to habitual choice. Our simulations show that this transition emerges as the optimal solution to the trade-off between policies that maximize reward or extrinsic value (habitual policies) and those that also consider the epistemic value of exploratory behavior (deliberative or epistemic policies)—the latter requiring VTE and the retrieval of episodic information via forward sweeps. We thus offer a novel perspective on the optimality principles that engender forward sweeps and VTE, and on their role on deliberate choice.

Substantial evidence indicates that animal behavior is determined both by deliberative processes (i.e., based on predictions of future outcomes and rewards) and by habitual reflexes (i.e., based on stimulus–response associations; Balleine and Dickinson 1998). The former are more resource intensive and sensitive to changes in task contingencies, while the latter are cheaper but inflexible; hence whether it is optimal to call on deliberative or habitual choice depends on the trade-off between the advantage of flexibility and computational costs (Balleine and Dickinson 1998; Dolan and Dayan 2013; Lee et al. 2014). In this paper, we try to understand the contextualization of behavior and the trade-off between deliberative and habitual choice from first principles, using Active Inference and Markov decision process models of exploitation and exploration (Friston et al. 2013, 2014, 2015; Pezzulo et al. 2015).

We focus specifically on vicarious trial and error (VTE) behavior, which is considered a hallmark of deliberation (Muenzinger 1938; Tolman 1938, 1939). This is based on the observation that, when rodents have to remember or search the correct route to a reward in a maze (e.g., a T-maze), they sometimes stop at choice points, to look left and right before choosing which direction to go. This has been interpreted as a signature of cognitive search and deliberation between the two choices (i.e., going right or left). In keeping with a role of VTE behavior for deliberation, it occurs early in learning and decreases or disappears after significant experience (Tolman 1939; van der Meer and Redish 2010; van der Meer et al. 2012) but it can increase again when task contingencies change (Blumenthal et al. 2011; Schmidt et al. 2013; Regier et al. 2015). VTE behavior has been consistently linked to hippocampal function (Hu and Amsel 1995; Voss et al. 2011; Regier et al. 2015). During deliberative choice and VTE, electrophysiological measurements of the hippocampus show that activation in place cells, which usually code for the current position of the animal, sweeps forward by recruiting first cells that code for one branch of the maze, and then cells coding for the other, thus transiently (and serially) coding for possible future routes. Simultaneously, covert expectations of rewards can be measured in the ventral striatum (Johnson et al. 2007; Johnson and Redish 2007; van der Meer et al. 2012). The hippocampus–ventral striatum pathway might represent a neurophysiological mechanism for VTE-based spatial deliberation and search processes, where memories of past experiences, associated with each choice, are recalled—or future events are simulated based on episodic memories—and used off-line to update the subjective value of the subsequent choice and select among plans (Lansink et al. 2009; van der Meer and Redish 2010; Pennartz et al. 2011; Pezzulo et al. 2013, 2014).

In sum, VTE behavior and associated hippocampal forward sweeps have been linked to deliberation and the (covert) evaluation of choice alternatives. They are an ideal paradigm for studying the various trade-offs between habitual and deliberate forms of choice. Indeed, their disappearance after a few trials, when the animals become familiar with the task contingencies, potentially marks a transition from deliberative to habitual forms of choice; and their reappearance when task contingencies change potentially marks the opposite transition from habitual to deliberative strategies. Most current models link VTE behavior to a form of model-based search among alternatives, but diverge in the specific mechanisms. For example, if VTE is involved in the calculation of value of the alternatives (“value-of-alternatives” theory) it should increase when the options are more difficult to differentiate. Alternatively, if VTE is responsible for flexible changes of strategies (“flexibility” theory), it should increase when behavior needs to be more flexible to achieve outcomes, see Pezzulo et al. (2013) and Regier et al. (2015) for discussions of these and related theoretical proposals. Here we advance a novel view that implies VTE behavior and associated forward sweeps in the (optimal) solution of the trade-offs between deliberate and habitual choice, which arise under various levels of extrinsic value, epistemic value, and uncertainty.

In a series of previous papers, we have described a Bayesian formulation of foraging behavior, known as Active Inference (Friston et al. 2015), in which agents maximize simultaneously “extrinsic value” (e.g., expected reward) and “epistemic value” (e.g., information gain or the resolution of uncertainty implicit in exploration or curiosity).^4^ In this paper, we use this Active Inference scheme to consider an important empirical phenomenon underlying VTE behavior; namely, the interruption of ongoing behavior to perform an epistemic action—the retrieval (or construction) of episodic memories through internal rehearsal (or simulation). Within this framework, we illustrate why this VTE processing disappears as the agent (e.g., rat) becomes more familiar with the task contingencies. Our formulation enables us to reduce VTE and its properties to the normative principles Active Inference, thus formalizing the computational mechanisms and functions of VTE.

We start from the idea that behavior can be controlled in different “modes,” habitual or deliberate, which we associate here to policies that follow stereotyped patterns to maximize reward or extrinsic value (habitual policies) versus those that also consider the epistemic value of exploratory behavior and thus perform epistemic actions (deliberative or epistemic policies)—the latter requiring VTE and the retrieval of episodic information via forward sweeps. More specifically, here forward sweeps constitute an “internalized (epistemic) action” that retrieves episodic memories, and uses them in a constructive manner to mentally simulate future (expected) episodes, analogous to constructive episodic memory processes in humans (Schacter et al. 2007; Hassabis and Maguire 2009; Buckner 2010). In keeping, here we model forward sweeps as covert (mnemonic/constructive) actions that are treated in exactly the same way as overt epistemic actions (e.g., exploratory actions), except that they sample cues or outcomes from memory and not the external environment. Importantly, in this formulation forward sweeps can become an explicit component of a policy or sequence of behaviors—hence the term “epistemic policies” for those policies that include forward sweeps. This means the agent has to evaluate policies that are mixtures of actions, some of which are covert (episodic retrieval) and others that are expressed overtly through moving to a new location. This formulation creates the interesting problem of balancing the epistemic value of covert mnemonic retrieval of construction, against the pragmatic value of securing a reward in the shortest possible time, that is, a subtle form of the exploration–exploitation dilemma (see the Discussion for a clarification of the links between “internal or covert” and “external or overt” exploration processes).

We will discuss how the (Bayes-optimal) scheme used here can be adopted to interpret two different forms of memory classically separated in the literature; namely, procedural and episodic memories. These two forms of memory correspond to different mechanisms within the proposed framework. Indeed, in the inferential scheme used below, “posterior beliefs” about the context or current environment (e.g., reward locations in a maze) become “prior beliefs” for the next round of exploration—or in other words, an agent can accumulate knowledge about its environment (e.g., reward location) in a series of trials and use this knowledge in the next trials. This form of (Bayesian) belief updating (between trials or sequences of behavior) can be conceived as an implicit or “procedural” form of memory—in terms of the evidence accumulated for one particular context relative to another, which in animal behavior links to (slowly) accumulated neocortical changes (McClelland et al. 1995) and to the gradual formation of action chains and habits in dorsolateral striatal areas (Graybiel 2008). With repeated exposure to the same environment, agents become increasingly confident about the best policies and—based on their acquired procedural knowledge—are able to engage in exploitative behaviors more efficiently and can “transfer” control to habitual processes. This contrasts with the initial exposure to the environment, when confidence about contingencies is low. It is in this case that search processes and episodic memories—associated with hippocampal functioning (Tulving 1972; Eichenbaum 2000)—may play an essential role, see Lengyel and Dayan (2008) for a discussion of the advantages of episodic control over other forms of control when an agent only has limited experience. We assume here that episodic memories concern the outcome experienced on the previous trial(s) and are used to construct or simulate future episodes on the fly (Hassabis and Maguire 2009), that is, when the agent is deliberating “within” trials—although experiences can also be “replayed” “between” trials for learning purposes and beyond (Sutton 1990; Diba and Buzsáki 2007; Pezzulo et al. 2014).

At the neurobiological level, we associate this process with hippocampal forward sweeps (Johnson and Redish 2007; van der Meer and Redish 2010; Pezzulo et al. 2014). Our primary question was whether the interplay between implicit (procedural) and explicit (episodic) memory could explain the disappearance of simulated forward sweeps that is seen empirically when rats become more confident with the contingencies of their environment. Heuristically, one can imagine that retrieving past outcomes (episodic memories) has epistemic value, which more than compensates for the delay in reward delivery (due to VTE and deliberation time) and the (putative) cognitive cost of episodic constructions. However, this will only be the case when there is a relatively high degree of uncertainty about reward locations or the current context. As experience with a particular environment is accumulated (through implicit or procedural memory) the epistemic value of episodic retrieval will decline. This would suggest a subsequent decrease of “covert” epistemic actions (associated with episodic memory retrieval and VTE behavior) and a gradual passage from a deliberate to a habitual mode of behavior.

To sum up, the novelty of this proposal is that we associate VTE behavior and hippocampal sweeps to (internalized) epistemic actions that can be part of the animal's behavioral plan. At decision points, candidate plans (policies) are evaluated and those that include VTE behavior (epistemic policies) are preferred when epistemic value prevails over extrinsic value. In this perspective, a transition from deliberate to habitual choice emerges as the optimal solution to the trade-off between policies that maximize reward or extrinsic value (habitual policies) and those that also consider the epistemic value of exploratory behavior (deliberative or epistemic policies)—the latter requiring VTE and the retrieval of episodic information via forward sweeps.

The proposed theory makes the prediction that VTE behavior depends on balance between epistemic and extrinsic value during a choice. In turn, epistemic value has a multidimensional sensitivity to uncertainty about the choice context (e.g., the reward contingencies) and the demands of behavioral flexibility (e.g., the need to track context changes to secure a reward). Thus, forward sweeps at decision points should be curtailed as an agent becomes more confident about the task contingencies (as in these occasions a stereotyped, habitual policy is sufficient), and be reinstated (for example) when task contingencies change and behavior needs to be flexible to adapt to the novel contingencies. This aligns our proposal with flexibility theory of VTE (Regier et al. 2015) but links the proximal mechanisms of flexibility to epistemic policies, which in turn depends on the (increased) epistemic value of a choice situation. By highlighting a role of epistemic value in engendering VTE, our novel proposal can generate additional predictions. For example, epistemic value is key in so-called “costly” choices, when an accurate estimation of the context is necessary to secure a reward and a wrong choice implies a “cost” such as long delay in reward consumption (van der Meer et al. 2010; Gupta et al. 2012), less so in conditions in which extrinsic value is plentiful and exploration redundant—and it is in the former, not the latter situations that VTE behavior should be observed.

To test this set of hypotheses, we performed simulations in a T-maze and a multiarm radial maze in which one arm was baited. We equipped agents with the prior belief that they were in a mildly volatile environment in which the maze (reward location) could change—and then allowed it to explore the maze over multiple trials (with occasional changes in the reward location). This prior belief is implemented by including in the agent's internal model a (small) expected probability that the context will change after each trial, see Table 1. Because the agent expects a degree of volatility a priori, its implicit (procedural) memory, due to Bayesian belief updating between trials, has residual uncertainty, especially in the early trials. By endowing agents with policies that include covert actions (episodic retrieval), we hypothesized a resolution of this uncertainty during early exploratory behavior that called upon forward sweeps to retrieve episodic memories. Furthermore, we hypothesized a resurgence of VTE behavior and forward sweeps in situations that require more flexibility, such as when contingencies change. Crucially, these are all emergent behaviors under ideal Bayesian assumptions—or in other words they emerge as optimal solution when an agent tries to maximize extrinsic and epistemic value and needs to rationally arbitrate between them.

**Table 1.**
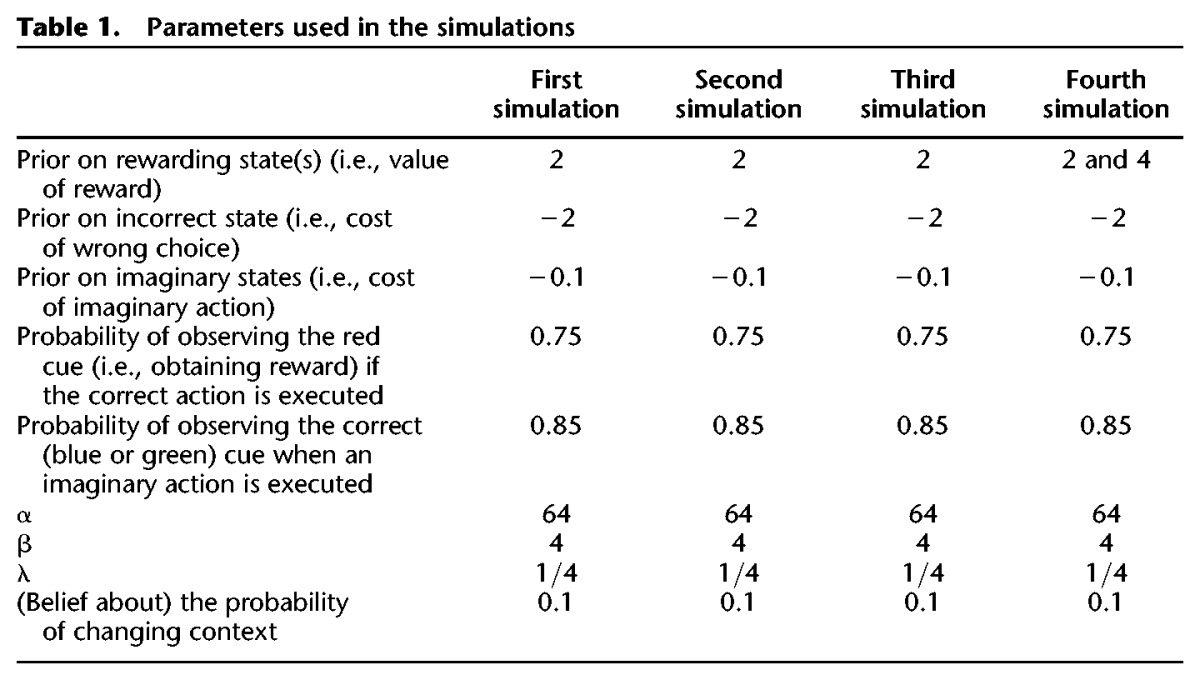
Parameters used in the simulations

## Results: VTE simulations using Active Inference

This section describes four simulations of VTE behavior using a Bayesian (Active Inference) formulation of choice and foraging (Friston et al. 2013, 2014, 2015). Our hope was to reproduce VTE behavior (and its neurophysiological underpinnings)—and then test the hypothesis that VTE-like processing would disappear as the simulated agents became familiar with the contingencies of the task at hand.

The mathematical methods used for the simulations are provided in the Materials and Methods section. Here, it is sufficient to say that the task of the agent is to select among a series of sequential policies (π), comprising action sequences such as: “go right, then go right,” “go left, then go left,” “perform VTE, then go right,” and so on. To this aim, the (*Q*) values of these policies are calculated, by summing their “extrinsic value” (i.e., the expected reward they deliver) and “epistemic value” (i.e., the information gain associated to its actions) over time points in the future. Formally, this (*Q*) value corresponds to the expected (negative) free energy of a policy, at any point in the future, as given by the following equation (for a full derivation of the equation, please see Friston et al. (2015):

$$\begin{array}{r} Q_{\tau }(\pi )=E_{Q(o_{\tau },s_{\tau }|\pi )}[\ln P(o_{\tau },s_{\tau }|\pi )-\ln Q(s_{\tau }|\pi )]\\ =E_{Q(o_{\tau },s_{\tau }|\pi )}[\ln Q(s_{\tau }|o_{\tau },\pi )+\ln P(o_{\tau }|m)-\ln Q(s_{\tau }|\pi )]\\ =\underset{\mathrm{Extrinsic}\;\mathrm{value}}{\underbrace{E_{Q(o_{\tau }|\pi )}[\ln P(o_{\tau }|m)]}}+\underset{\mathrm{Epistemic}\;\mathrm{value}}{\underbrace{E_{Q(o_{\tau }|\pi )}[D[Q(s_{\tau }|o_{\tau },\pi )||Q(s_{\tau }|\pi )]]}}. \end{array}\;\left(1\right)$$

Intuitively, the *Q* value of a policy sums up the value of all the observations that the agent (should) gather by following the policy from start to end—including most prominently reward observations (note that, in our simulations, all the policies have the same length). Mechanistically, it is calculated by starting from the agent's belief about its present state (which can be uncertain), by propagating predictions about future states that are expected to be visited given the policy, and by considering the value of the outcomes that can be (probabilistically) obtained in these states.

This evaluation, in turn, requires equipping the agent with three types of a priori knowledge: a generative model (m) of the task, which essentially encodes the contingencies between the actions prescribed by the policies and the resulting states (s) and observations (o); beliefs about the agent's current state; and beliefs about the extrinsic value of the goal (rewarding) state(s).

The evaluation assigns policies a value depending on two components that—as we will see—need to be balanced: extrinsic value and epistemic value. The extrinsic value corresponds to delivering rewarding outcomes, e.g., a rewarded branch of a T-maze. In this Bayesian formulation, the animal's goals and needs are expressed in terms of prior probabilities or preferences for particular (rewarded) outcomes, and policies that minimize (in probabilistic terms) the distance^5^ between the current state and the goal states (i.e., states in which the preferred outcomes can be obtained) have thus high extrinsic value.

However, the evaluation also considers the epistemic value or information gain furnished by new information that disambiguates among hidden states (e.g., the location of an unseen reward). In this perspective, a policy that permits an agent to estimate in which choice context it is (i.e., what are the task contingencies) has high epistemic value. Of note, all the policies that we will use in the simulations have some epistemic value, because obtaining (or not obtaining) a reward is per se informative about choice context. However, some policies also include epistemic actions that correspond here to a covert retrieval of episodic memory and VTE behavior. Selecting policies that include epistemic actions and VTE has short-term costs (e.g., might delay reward acquisition) but can also have long-term advantages. Our simulations will show in which cases policies that include, or not include, epistemic actions are preferred.

Once the agent has calculated the (*Q*) value of the policies according to Equation 1, it selects one using a precision-controlled *softmax* rule (i.e., a choice rule that assigns policies a probability to be selected proportional to their *Q* value, and includes a parameter that regulates exploration depending on the estimated reliability or precision of the policies, see later). The agent then executes the current action prescribed by the sequential policy, does a transition to a new state, samples probabilistically the observation available in this state (e.g., a reward observation), and based on this observation updates its belief on its current state and context. At this point, the evaluation starts again, in order to select the second action to take, and so on; this implies that an agent can start acting based on a given policy, and then select a new policy along the way based on the new observations it obtains. See Materials and Methods for further details.

### Summary of the four simulations

**Figure 1.**
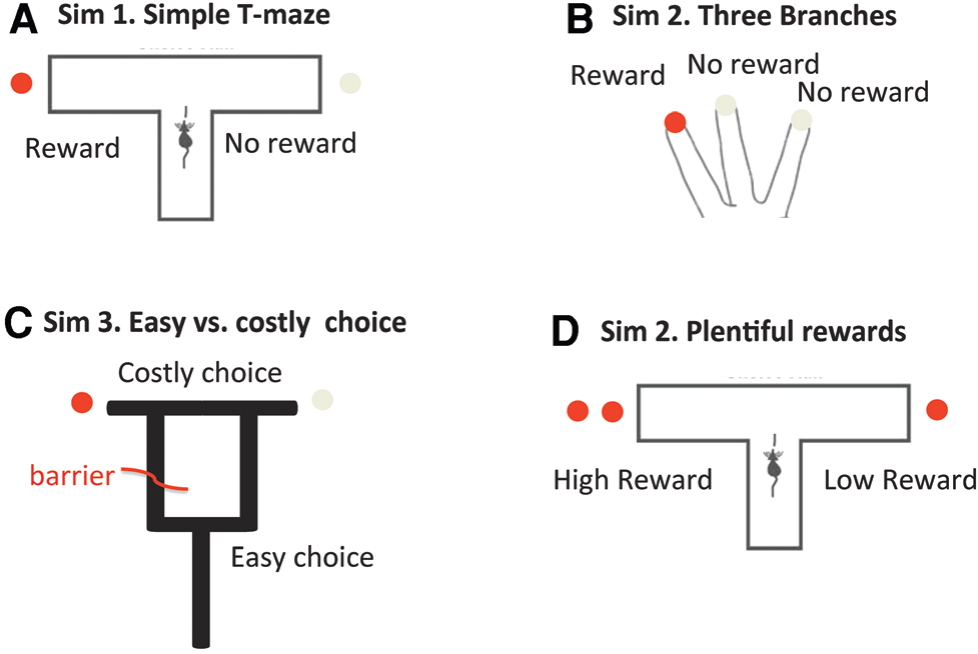
Setups of the four simulations. (*A*) First simulation, with two branches. (*B*) Second simulation, with three branches. (*C*) Third simulation, with two consecutive choices, easy and costly. This example shows a sample configuration that corresponds to the right–left context, where the path to the left is blocked. The other three possible maze configurations are left–left, left–right, and right–right. (*D*) Fourth simulation, with two branches, which can be associated to high, normal or no reward.

The setups (T-mazes and radial mazes) used in the four simulations are shown in Figure 1. In the first stimulation, we address the choice between two branches of a T-maze, one of which is rewarded, of the kind where evidence on vicarious trial and error (VTE) and forward sweeps has been recently reported (Johnson and Redish 2007; van der Meer and Redish 2010; Pezzulo et al. 2014). In this simulation, the rat is uncertain about two possible contingencies (i.e., whether the reward location is to the right or left), which—in terms of the agent's model—correspond to two hidden contexts. In this setting, we will see that the rat performs VTE behavior—but only in the initial trials, until uncertainty about the context resolves with experience, thus making a transition from a deliberate (VTE) to a more habitual form of choice. We will also see that, when the task contingency (aka context) changes, VTE behavior is reinstated, suggesting that it (only) plays a role when the animal's behavior needs to be flexible.

In the second simulation, we use a maze with three branches. Here, there are three possible contingencies (or contexts), corresponding to finding rewards to the left, central, or right branch of the maze. While in the first simulation sampling either branch is equally informative about the current context, in the radial maze the rat has to “probe” each branch individually—or more precisely, all but one branch—to infer the context it is in.

In the third simulation, we model a more complex decision situation: a double T-maze, with two consecutive decision points (this is similar to the setup of van der Meer et al. 2010; Gupta et al. 2012, but includes two rather than four decision points). The first decision point is often referred to as “easy choice,” while the last choice is considered to be a “costly choice”—and it is only in these “costly” decision points that VTEs are observed (van der Meer et al. 2010; Gupta et al. 2012). What makes the early decision points “easy” is the fact that the animal can easily and quickly recover from a wrong choice; in our simulations, these decision points do not lead to absorbing states and so even if the animal selects the wrong branch, it can successively select a policy that leads to reward. What makes the last decision point “costly” is the fact that a wrong choice in this point would significantly delay reward consumption, because basically the animal has to come back to the home location of the maze and do all the choices again before having the chance of obtaining a reward. In our simulations, (only) costly decision points mark transitions to absorbing states, and so if an animal selects the wrong branch it cannot obtain reward in this trial.

In the fourth simulation, we return to the maze with two branches of the first simulation, but expand the number of task contingencies (or contexts) by introducing scenarios in which the reward can be in both locations, but one branch delivers more reward (or with a higher probability)—thus introducing a choice between a high versus low-reward choice. This simulation highlights the context-sensitivity of VTE behavior and its alleged dependence on the relative extrinsic and epistemic reward.

In summary, these simulations are intended to illustrate the role of VTE and epistemic actions in a normative model of foraging, and the ways they change depending on the task contingencies and experience.

### First simulation: a simple T-maze simulation

#### Setup

The scenario that we consider in our first simulation is a T-maze with two branches; see Figure 1A. The goal of the agent is to find a reward site, which can be to the left or right, depending on the context (which is initially set to “reward to the left,” but changes during the simulation). The agent has to select among multiple policies, some of which go directly to the right or left (which are absorbing states), whereas others include some forward sweeps, followed by overt movement to the right or left.

The agent is initially uncertain about the context: after each choice, procedural knowledge (of the task contingencies) is updated in a Bayesian manner (Friston et al. 2013, 2014, 2015), so that the rat becomes increasingly confident about its choices. At the same time, we endowed the agent with the prior belief that the environment is volatile and can change (with a small, fixed probability); this is essential for the agent to update its belief about the context (reward location) when, as we will see below, we change it during the experiment.

#### The generative model

**Figure 2.**
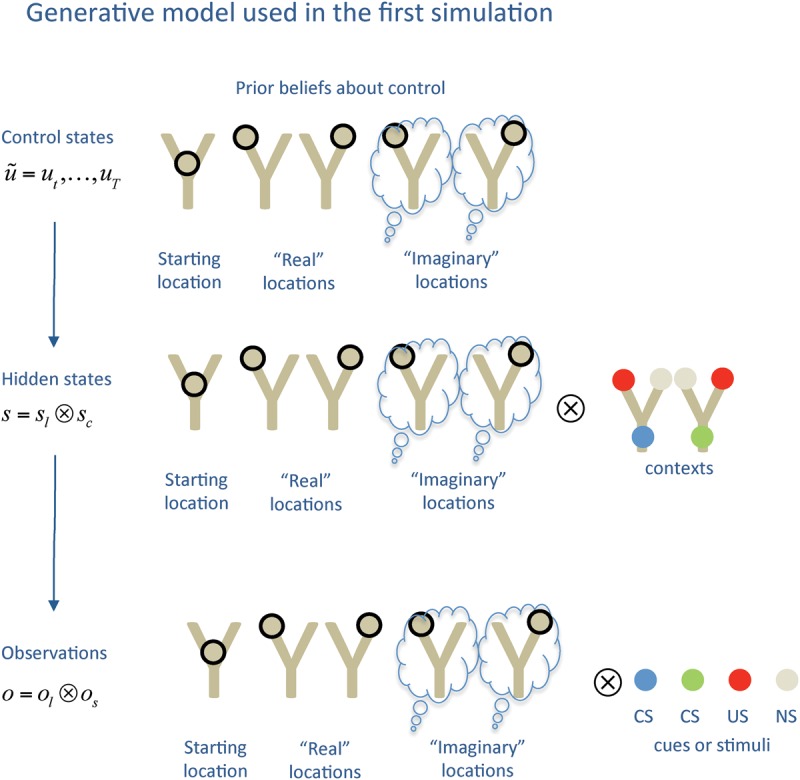
Generative model used in the first simulation, which includes control states, hidden states, and observations, along with their contingencies. The symbol ⊗ denotes a Kronecker tensor product. See main text for explanation.

The agent's generative model is shown in Figure 2. It includes 10 “hidden states” (5 locations × 2 contexts) and 20 “observations” (5 locations × 4 cues or stimuli). The two contexts correspond to the two reward contingencies: “reward is on the left” or “reward is on the right.” The five location states correspond to three locations of the maze (center or start location, left arm, and right arm) plus two additional “imaginary” or mnemonic locations that correspond to episodic memories of the right or left arm. Visiting these “imaginary” locations does not correspond to an overt physical action but to a covert and deliberative VTE event; i.e., a forward sweep to the right or to the left, respectively. Here, we treat these imaginary locations as hidden states (by analogy with real spatial locations) because, technically, the animal can decide whether or not to “visit” them covertly. The transitions along the hidden states are controlled by five control states, which effectively move the rat to each of the five locations, within each context. Finally, the model includes four outcomes or cues: red (reward), white (neutral), blue and green (contextual cues which correspond to the episodic recall of a reward outcome on visiting a given branch of the maze); see also Friston et al. (2015). As in many animal experiments, all these cues are delivered probabilistically. The reward cue is delivered 75% of the time in the correct branch of the maze (otherwise, the mutual cue is delivered). Similarly, the correct contextual (blue or green) cue is delivered 85% of the times in the corresponding context (otherwise the alternative contextual cue is provided). In other words, even if the rat is certain about the reward location, its retrieval is imperfect.

It is important to note that, by visiting a state, the agent collects cues or observations that can help disambiguate the context it is in. For example, if it visits a “left” control state and collects a red cue (reward), it can infer that it is in a context where “reward is delivered to the left.” By accumulating this information, the agent can successively choose “left” with higher confidence. This marks an important difference with associative learning theories, because—in Active Inference—behavior is not selected based on (learned) stimulus–response or action–outcome associations, but rather through a (Bayesian) inference that selects policies based on beliefs about context (see Equation 1).

A second important point to note is that, in this scenario, visiting an “imaginary” state corresponds to performing VTE (and implicit forward sweeps) to a given branch. During this covert behavior the agent remembers or simulates a previous reinforcing (or neutral) episode; i.e., it covertly retrieves from memory (or simulates) experiencing one of the two contextual cues (blue or green). This form of episodic memory or simulation is not considered here to be a rewarding experience per se—on the contrary, we associate states that are “visited” during VTE with a small prior cost (e.g., the cost of forward sweeps in terms of time and cognitive resources). In the Active Inference framework, costs (e.g., cognitive and metabolic costs) are absorbed into the probability model (Friston et al. 2012b); this means that costly states (here, imaginary states) have an a priori low probability in the generative model and thus—all other things being equal—are less likely to be visited. However, there are circumstances when this prior cost is more than offset by the value of reaffirming the current context, before committing to a choice. In other words, here VTE and forward sweeps have a cost in terms of extrinsic value, but have epistemic value as they afford new information (blue or green cues) that can inform state (context) estimation and future choices. This formulation thus allows us to compare policies that lead the animal directly to rewards (which we associate with habitual forms of choice) and policies that firstly exploit epistemic actions and “imaginary” states (which we associate with deliberative forms of choice and covert exploration).

#### Simulation

The simulation was integrated over 20 trials. The agent starts from the center (home) location. Initially (in trial 1) the agent has a flat (uninformative) prior belief about the context or reward location; this belief is updated as described in Friston et al. (2015). On each trial, the agent has to select one of its policies based on its beliefs about context, knowledge of the task contingencies (as encoded in the generative model that links hidden states to observations). This selection is effectively an inference about sequential policies that maximize (the path integral of) value that is based upon prior preferences or utility.

In Active Inference, utilities are encoded in terms of Bayesian “priors” over future outcomes, and the agent believes that the most likely policies minimize the divergence between expected and preferred states, encoded in these priors. These priors are usually specified in terms of log probabilities or utility. Intuitively, positive utility corresponds to reward, while negative values correspond to cost. In this simulation, the correct (rewarding) arm of the maze is assigned a high utility, the wrong (unrewarding) arm of the maze is assigned an equivalent negative utility, and the imaginary states are assigned small negative values, so that there is a cost for performing forward sweeps. See Table 1 for the value of the parameters used in this and subsequent simulations.

The policies selected by the agent comprise sequences of three control states, and can include either “real” control states (e.g., go to the left arm), which lead the agent to absorbing hidden states (i.e., once the animal has reached the left arm, it can only select another “left arm” control state) or “imaginary” control states (i.e., remember going to the left arm), which lead the agent to hidden states that are not absorbing. Here, for simplicity, we consider 14 possible policies. These include one “imaginary” control state (e.g., simulate going left, then go left), two “imaginary” control states (e.g., simulate going left, simulate going right, then go right), or no imaginary control states (e.g., go left).

Selecting one or two “imaginary” actions or control states (i.e., performing one or two forward sweeps) enables the agent to recall one or two episodes, and thus garner more (mnemonic) cues for committing to a choice. At the same time, performing forward sweeps has a cost for the animal, because it implies a delay in reward consumption and/or the expenditure of metabolic or cognitive resources. In the model, this cost is encoded as a (small) negative utility associated to the imaginary hidden states (which translates into a lower probability in the generative model). The interesting comparison here is between the policies that do and do not include, “imaginary” actions, as the former correspond to VTE and the execution of forward sweeps.

#### Results

**Figure 3.**
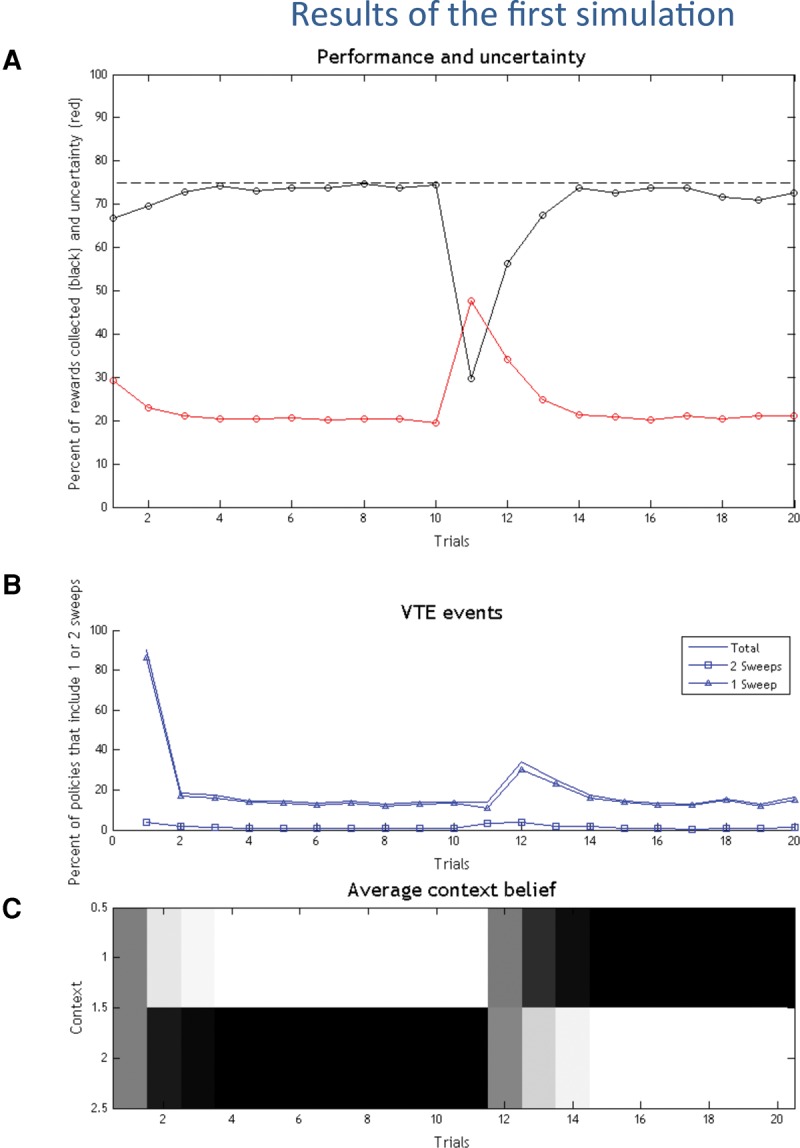
Results of the first simulation. (*A*) Performance (black) and uncertainty (red) in the 20 experimental trials. Performance is measured as the percentage of correct responses, with the dashed horizontal line representing the highest possible performance. Uncertainty represents the agent's uncertainty about the current context. (*B*) VTE events, shown as the percentage of selected policies that include imaginary actions/forward sweeps (blue). See main text for explanation. (*C*) Agent's belief on its current context (white is high, black is low).

The results of the first simulation are shown in Figure 3. Here, we show the performance of the rat (black), its uncertainty (red) and the number of VTE forward sweeps, over 20 trials. The results shown here and in the following simulations are an average over 1000 runs of the same model. Note that, although the model is always initialized in the same way, policy selection and the sampling of outcomes are stochastic processes.

Figure 3A shows the performance (i.e., rewards collected) and uncertainty of the agent over the 20 trials. Figure 3B shows the number of times that the agent uses a policy with one or more imaginary actions or forward sweeps. The agent only uses VTE in the first trials, and stops doing so after few trials, marking a transition from deliberative VTE to a more habitual form of choice. Here, the preferred policies are those that include a single forward sweep (compare with the second simulation below). During trial 11, we inverted the context (i.e., the reward contingencies), from “reward to the left” to “reward to the right.” It is at this point that the agent's performance suddenly decreases, because it acts on the basis of a false belief. In turn, this failure causes a second transition, from habitual to deliberative choice, as at the next trial, the agent starts using VTE again.

This behavior can be further analyzed by considering the belief about the reward context, which is shown in a black-to-white (low-to-high) scale in Figure 3C. After just one trial, the agent becomes very confident about its context. The belief becomes more uncertain after the tenth trial, but only transiently, when the agent realizes that the context has changed.

#### Discussion

In this first simulation, we replicated the experimental finding that rodents engaged in T-mazes use VTE/forward sweeps in the first few trials only, and then exhibit a more habitual form of behavior. Furthermore, VTE/forward sweeps can be reinstantiated by changing the reward contingencies (Johnson and Redish 2007; van der Meer et al. 2012; Schmidt et al. 2013; Pezzulo et al. 2014). In our simulations, changing the model parameters makes these transitions faster or slower in terms of number of trials that involve or do not involve VTE, but not the overall pattern of results (results not shown).

Crucially, we have replicated these results by appealing only to Bayesian inference, not a set of stimulus–response or action–reward associations. This Bayesian perspective provides a normative explanation for the balance of information gain and utility maximization—that is, exploration–exploitation (Friston et al. 2015). It suggests that the balance of different choice strategies, deliberative or habitual, can be explained in terms of normative principles; specifically, as the utility of extrinsic rewards relative to epistemic value provided by covert action or deliberation. Only when there is no more epistemic value (e.g., because the animal has resolved its uncertainty about the current context) can forward sweeps be suppressed.

By the same token, we show that the balance between deliberative and habitual choice strategies can be cast within a single Active Inference scheme, which supports epistemic actions. This view can be compared with alternative schemes based on multiple controllers that operate using different (model free and model based) reinforcement learning principles and require an external arbitrator (Daw et al. 2005; O'Doherty et al. 2015). Here, the key point is that Active Inference simultaneously considers two forms of value: extrinsic value and epistemic value. It is when the former surpasses the latter that behavior can be habitized. Policies that try to maximize directly extrinsic value using stereotyped responses and without performing epistemic (or exploratory) actions can be considered more habitual. These are indeed successful when the environment is unambiguous—or, in other words, familiar environmental cues or the animal's current belief state unambiguously specify the best policy. When this is not the case, epistemic actions (here, covert samples of episodic information from memory) must be performed before committing to a course of action. Empirically, it is only in these latter conditions—which we associate to deliberative choice—that VTE behavior and forward sweeps are observed. This simulation illustrates that forward sweeps are indeed executed in the initial trials, or when contingencies change; that is, when the agent is uncertain about its current context and behavior needs to be flexible to secure a reward.

In short, all the behavior simulated under active inference is quintessentially model based. The key distinction between deliberative and habitual responses can then be reduced to a distinction between policies that entail epistemic actions and policies that do not. Here we link epistemic actions to a form of “mental” exploration, in which episodic content is retrieved from memory (Eichenbaum 2000; Tulving 1972) or simulated/recombined in a constructive way to construct novel episodes (Hassabis and Maguire 2009; Schacter and Addis 2007), thus leaving open the possibility that VTE-based declarative control engages a mixture of model based and episodic components. In turn, the episodic component can include a mixture of contextual, spatial, and reward information (e.g., through reactivations of hippocampus and ventral striatum, Lansink et al. 2009). The engagement of epistemic behavior does not require any special arbitration; it is a natural consequence of casting the utility of a reward in terms of a prior belief, which enables it to be compared directly with the epistemic value of (overt and covert) exploratory behavior. This is a key simplification afforded by Active Inference, enabling habitual and deliberative planning to be treated within the same normative framework and, potentially, implemented within the same distributed neuronal systems.

Finally, it is also worth noting that the reason the agent changes its belief (rapidly), when contingencies change, is that we endowed it with the prior belief that the reward contingencies are volatile—and can change from one trial to another, although with a small probability. By removing the volatility prior, a trained agent would be less sensitive to changing contingencies. This idea resonates with related Bayesian theories of foraging and learning, in which animals make their choices based on the (learned) statistics of the environment, including higher order statistics such as volatility (Gallistel 2012). In this perspective, animals learn and track the statistics of the environment, at multiple levels, to adapt their behavior and learning rates in efficient ways (Gershman and Niv 2012; Gallistel and Matzel 2013; Pezzulo et al. 2013).

### Second simulation: radial mazes and resolution of uncertainty

#### Setup

One limitation of the simulations above is that there are only two possible contexts, and so collecting the cue of (say) “no reward to the left” automatically implies “reward to the right.” This is also why the agent only performs one sweep (Figure 3B), when deliberating. To make things more realistic, we now describe a second simulation, where the agent navigates a maze with three arms (see Fig. 1B). The possibility of three mutually exclusive contexts (reward to the right, center or left arm) means that probing each branch of the maze will necessarily increase epistemic value.

#### The generative model

The generative model used in this second simulation was the same as in the first, except that there are three rather than two contexts, and two additional control states: one additional true location, which corresponds to the third arm of the maze, and the associated imaginary location. The ensuing model thus comprises 21 hidden states (7 locations × 3 contexts). As in the first simulation, there are 4 cues, but now the number of possible observations is 28 (7 locations × 4 outcomes). As in the first simulation, the red cue (reward) can be (probabilistically) collected in only one of the arms, while the other two arms deliver white (neutral) cues. The “imaginary visit” of the three branches delivers a blue or a green cue, which correspond to the episodic recall of a rewarding or a neutral outcome.

In this simulation, we considered 48 policies, comprising sequences of four control states. As in the first simulation, here the interesting comparison is between policies that lead the agent directly to one of the three arms of the maze (which are absorbing states), and those in which the agent engages in one or more imaginary actions.

#### Simulation

The simulations covered 30 trials, where we changed the reward contingencies (and context) at trials 11 and 21. As in the first simulation, the agent always starts from the center (home) location with uninformative prior belief about the context (reward location), which is successively updated over successive trials.

#### Results

**Figure 4.**
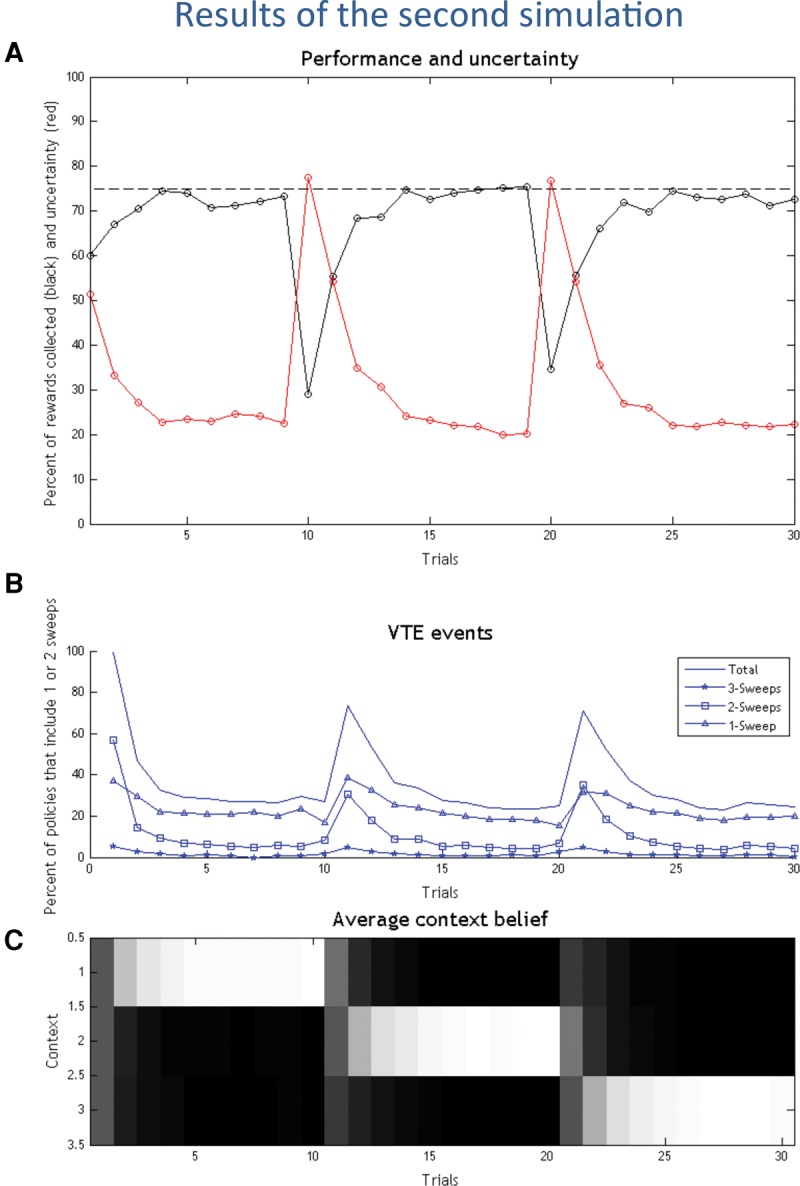
Results of the second simulation. (*A*) Performance (black) and uncertainty (red) in the 30 experimental trials. (*B*) VTE events, shown as the percentage of selected policies that include imaginary actions/forward sweeps (blue). See main text for explanation. (*C*) Agent's belief on its current context (white is high, black is low).

The results of the second simulation are shown in Figure 4. One can see that the same pattern of behavior emerges, with an initial rapid decrease of the VTE/forward sweeps marking the passage from deliberate to habitual forms of choice, and the reinstantiation of a deliberative strategy, when reward contingencies change.

#### Discussion

The analysis of context belief updating (Fig. 4C) confirms that the agent infers the right choice context correctly and rapidly, and then stops performing forward sweeps. A fine-grained analysis of the policies implying forward sweeps (Fig. 4B) shows that VTE is never completely extinguished, but declines rapidly. Furthermore, this analysis shows a preference for policies with one, or at most two, imaginary actions.

The same overall pattern of results can be obtained with different parameterizations of the model (results not shown). In particular, the partial or complete extinction of forward sweeps depends on the relative balance between extrinsic and epistemic value in each setup—this is a point that we consider in more detail in the next two simulations.

### Third simulation: easy versus costly choice

#### Setup

The scenario that we consider is a double T-maze, with two consecutive decision points; see Figure 1C. In principle, there are four possible paths (left–left, left–right, right–left, right–right); however, a barrier located after the first decision point blocks two of them. Importantly, a wrong decision in the first (easy) but not the second (costly) decision point can be undone, by selecting an appropriate policy. This is because only the states accessible from the second decision point are absorbing.

A similar setup (but with up to four decision points) has been adopted in a series of rodent studies (van der Meer et al. 2010; Gupta et al. 2012). Using the terminology of these studies, here we consider the first decision point to be an “easy choice” and the second decision point a “costly choice”—because a wrong choice at this latter point would significantly delay reward consumption (i.e., the animal has to traverse all the maze, come back to the home location, and start a new trial in order to get the chance again to obtain a reward).

#### The generative model

The generative model used in this third simulation was similar to the first, but there are four rather than two contexts, corresponding to the four choice possibilities (left–left, left–right, right–left, right–right) to be executed. Furthermore, there are 11 control states: one for each of the three possible decision points (indeed, a first, easy decision point is followed by one of two costly decision points, one on the left or one on the right), six imaginary states (two for each decision point, the former imaging the consequences of going left and the latter the consequences of going right) and two final states (the left return arm and the right return arm). The ensuing model thus comprises 44 hidden states (11 locations × 4 contexts). We introduced six contextual cues. As in the first simulation, the reward cue can be (probabilistically) collected in only one of the arms, while the other arm delivers (probabilistically) a neutral cue. The “imaginary visit” of the two branches of the costly choice delivers a blue or a green cue, which correspond to the episodic recall of a rewarding or a neutral outcome. The “imaginary visit” of the two branches of the easy choice delivers two distinct cues, yellow and black, which signal that the agent is in one of two contexts (yellow: left–left, left–right; black: right–left, right–right), thus partially reducing its uncertainty.

As we have mentioned, a first difference between the former (easy) and the latter (costly) decision is that states accessed from the latter (but not the former) decision point are absorbing. A second difference between the former (easy) and the latter (costly) decision is that, in our setup, performing imaginary actions in the former does not provide contextual cues that uniquely prescribe the correct behavioral policy. This is because the contextual cues that can be obtained (via epistemic actions) in the former decision point inform on the validity of the left versus right actions at this “easy” decision point, but not on the choice to be made at the “costly” decision point. This aspect of the model represents the fact that forward sweeps have limited span. Rather, the contextual cues that can be obtained (via epistemic actions) in the latter decision point function exactly as in the previous simulations.

The convergence of these two factors would improve the utility of performing forward sweeps in the presence of “costly” decision points, but it remains to be established if this prediction holds when one simulates the specific experimental details.

#### Simulation

The simulations covered 40 trials, where we changed the context (and reward contingencies) at trials 11, 21, and 31. As in the first two simulations, the agent always starts from the center (home) location with uninformative prior belief about the context (reward location), which is successively updated over successive trials. The context is changed every 10 trials using this sequence of contexts: left–left, left–right, right–left, right–right. As in the previous simulations, a critical comparison is between policies that include or not include VTE events. Here, however, another critical comparison is between policies that include VTE events at easy versus costly decision points. Note that for the sake of simplicity we only consider policies that allow one single VTE event per decision point (thus, we do not perform a comparison between policies that include one versus two VTE events).

#### Results

**Figure 5.**
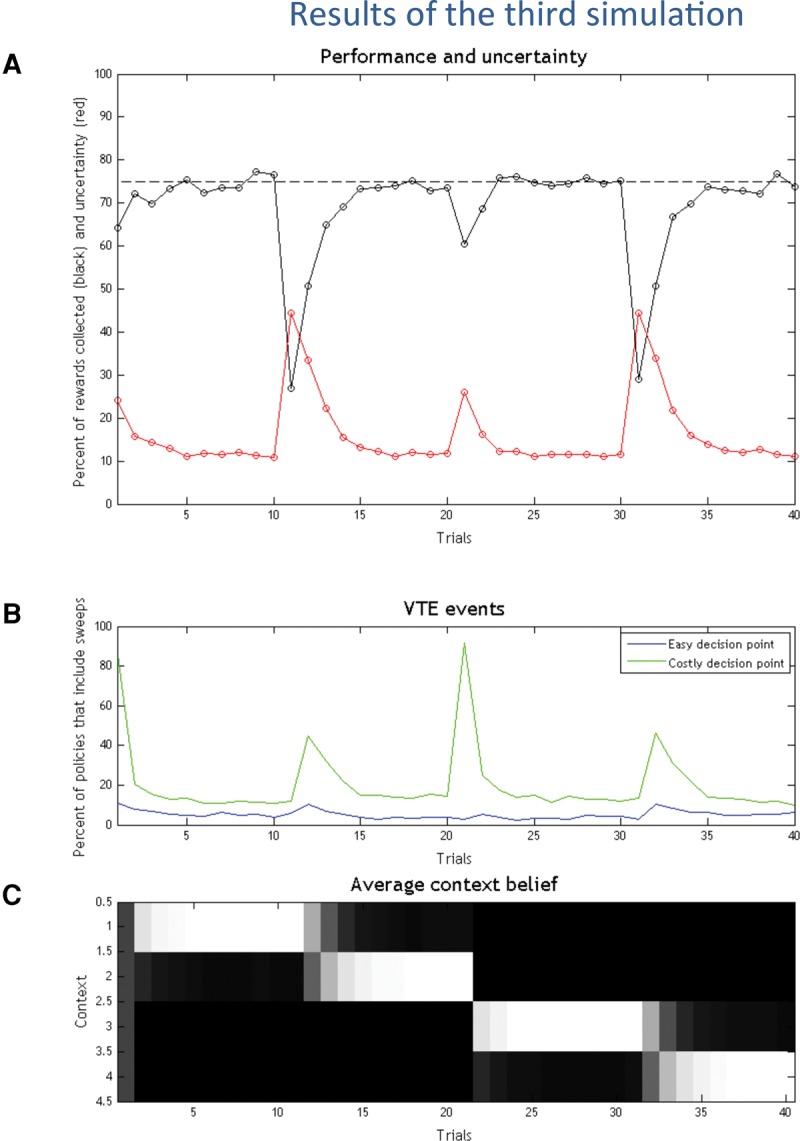
Results of the third simulation. (*A*) Performance (black) and uncertainty (red) in the 40 experimental trials. (*B*) VTE events, shown as the percentage of selected policies that include imaginary actions/forward sweeps in the easy (blue) or costly (green) decision points. (*C*) Agent's belief on its current context (white is high, black is low).

The results of the third simulation are shown in Figure 5. Here, like in the first two simulations, one can see marked increases and decreases of the VTE behavior/forward sweeps observed at the beginning of the experiment and when reward contingencies change. Importantly, the VTE/forward sweeps are almost exclusively present in the “costly” decision points, see Figure 5B.

#### Discussion

Coherent with the previous simulations, here VTE behavior and forward sweeps mark a transition from deliberate to habitual forms of choice—and associated habitual policies—which become available when the correct choice context has been estimated. The fact that VTE events are mostly limited to “costly” decision points is coherent with the literature (van der Meer et al. 2010; Gupta et al. 2012) and illustrates that it is at this point that epistemic actions are more efficacious to inform the correct choice and prevent a significant delay in reward consumption.

The higher peak after trial 21 marks an important transition between policies that differ in both the required actions (from left–right to right–left). Although this situation does not induce higher contextual uncertainty than the previous transition (from left-right to left-left), it requires a more flexible change of strategy—as one cannot keep executing the first (left) action as in the previous case. It is this case that demands more forward sweeps, in keeping with a recognized role of VTE behavior in promoting behavioral flexibility (Papale et al. 2012). Here, the increased number of VTE events is plausibly due to the fact that, after the first (left) choice, the animal faces the impossibility to pursue its policy—as the path is blocked. Hence, it is already at this point (after the first decision point of trial 21) that it knows that context has changed and needs to invoke a VTE event to flexibly change policy. This situation can be contrasted with all the other simulations, in which the agent cannot infer a change of context midway in a trial (here, trial 21), but only at the end of it, if it fails to collect an expected reward. Supporting this analysis is the fact that, in this simulation, context changes in trial 21 and VTE events occur mostly in the same trial; whereas in the other simulations, VTE events occur mostly one trial after context has changed (e.g., context changes in trial 11 and VTE events occur mostly in trial 12).

The fact that epistemic actions (which in our simulations have an associated cost) are rarely executed at the first (easy) decision point indicates that—besides being less informative—they are not necessarily required whenever uncertainty exists between alternatives. Rather, they are only demanded when the epistemic value they afford exceeds the extrinsic value of the overt behavioral policies—or, in other words, when a wrong choice would imply a significant cost or delay in reward consumption. In the latter simulation we study in more detail this latter phenomenon: the suppression of VTE behavior and forward sweeps when the value of the alternative behavioral policies is increased, thus exceeding any epistemic value.

### Fourth simulation: plentiful rewards

#### Setup and generative model

The fourth simulation introduces a choice scenario in which one or both arms of the T-maze are associated to rewards. Here, we compare explicitly two different situations. In the former situation, analogous to the first simulation, one branch is baited with reward and the other corresponds to a nonpreferred alternative (i.e., the baited arm has a “value” of 2 and the nonbaited arm of −2). As we have seen, this corresponds to a “costly” choice situation, in which making the wrong choice has an associated cost that surpasses the costs of epistemic actions. In the latter case, we associate rewards to both arms and make the value of one arm (called “high reward”) twice the value of the other (called “usual reward”); see Figure 1C. We are interested in the comparison between the former (costly) and the latter (plentiful) choice situations.

In this simulations, the 5 control states and the 14 policies are the same as in the first simulation. However, there are now four contexts or reward contingencies. The first two are the same as in the first simulation, when either the right or the left arm of the maze is rewarded. The last two correspond to the context when one arm of the maze (left or right) delivers the usual reward, while the other (right or left, respectively) affords a high reward (i.e., double reward). As we will see, when these two latter contexts are in place, any policy that goes directly to a rewarded branch (including the less-rewarded arm of the maze) can overcome the value of policies that include VTE and implicit forward sweeps. Finally, there are six cues. The first four are the same as in the first simulation, while the latter two (dark red and dark blue) correspond to the presence and the episodic retrieval of the high reward, respectively, analogous to the red and blue cues in the first simulation.

#### Simulations

The simulations covered 40 trials, where we changed the reward contingencies at trials 10, 20, and 30. The first two contexts introduced in trials 1 and 10 are the same as those used in the first simulation; however, at trials 20 and 30, we introduced the two new reward contingencies, in which the two arms of the maze deliver a normal reward and a high reward. As in the first simulation, the agent always starts from the starting (center) location with uninformative priors.

#### Results

**Figure 6.**
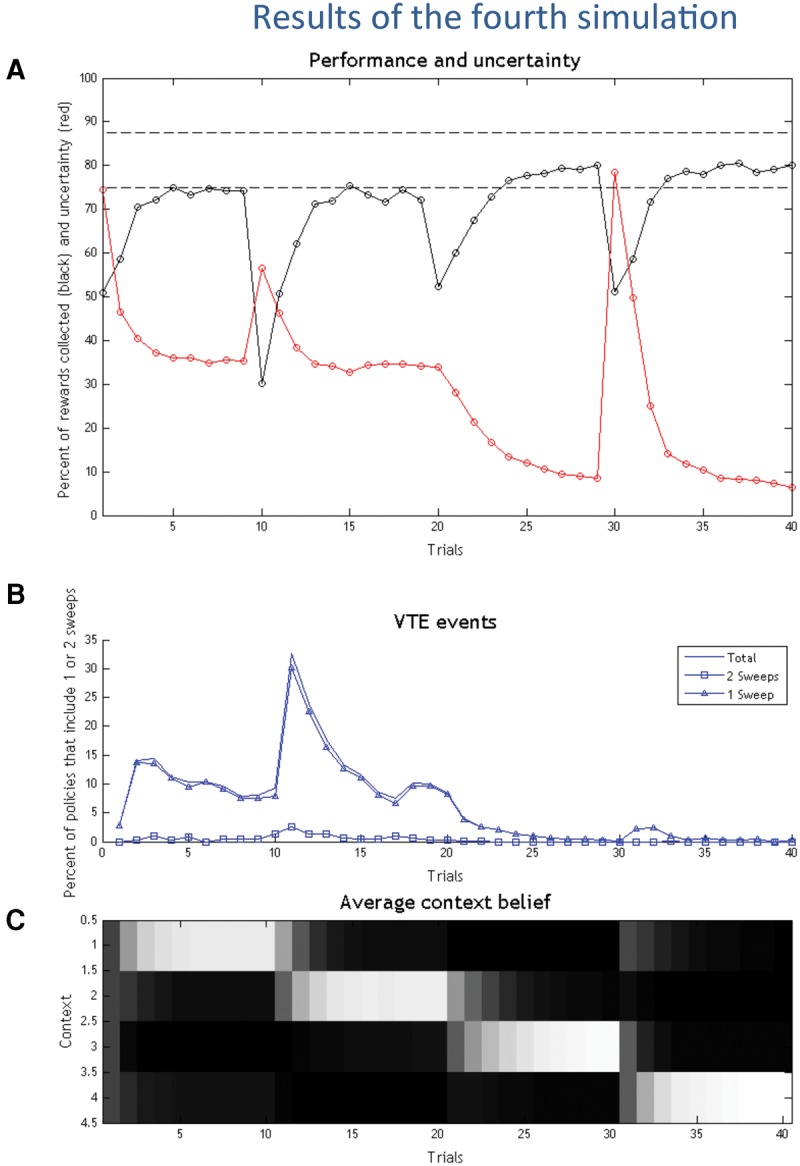
Results of the fourth simulation. (*A*) Performance (black), uncertainty (red), and percentage of selected policies that include imaginary actions/forward sweeps (blue) in the 40 experimental trials. Note that in this figure there are two dashed horizontal lines, representing the highest possible performance in the first half (lower line) and the second half of the simulation (higher line), respectively. (*B*) Agent's belief on its current context (white is high, black is low). (*C*) Detailed analysis of the policies that include zero, one, or two forward sweeps.

The results of the fourth simulation are shown in Figure 6. In the first half of the simulation (trials 1–19), the pattern of behavior is similar to that of the first simulation, with an initial use of epistemic policies based on forward sweeps, which are rapidly suppressed. Although the choice situation looks identical to the first simulation, there is an important difference: while in the first simulation the agent resolved uncertainty over two contexts, here it has to consider four contexts, two of which include rewards in both arms of the maze. Clearly, in this situation the value of taking overt action soon (versus covert action) is much higher, and this explains why the agent uses fewer forward sweeps, relative to the first simulation.

In the second half of the simulation (trials 20–40), in which reward is delivered in both arms of the maze, the forward sweeps are almost completely extinguished. Here, the key insight is that this pattern does not depend on a resolution of uncertainty (on the contrary, uncertainty is quite high around trials 30–31). Rather, the scarce use of forward sweeps is due to the fact that introducing abundant reward lowers the (relative) value of epistemic actions, because even an apparently “suboptimal” choice is more valuable than a forward sweep.

#### Discussion

The results of this simulation (especially the second half) illustrate that even if performing a forward sweep would reduce uncertainty about the context, the policies that entail forward sweeps have lower overall value (extrinsic plus epistemic value), even compared with policies that secure a low reward. This is because they include costly imaginary states, and delay reward delivery. To better understand this point, and the prevalence of extrinsic value in this simulation, note that all the policies have a higher value compared with the simulations above. This is evident in Figure 5A, which shows that the performance increases in the second half of the simulation. The reason is, of course, that both arms are rewarded. The situation here is markedly different from the first simulation, where the “wrong” arm of the maze is more costly than imaginary actions.

It is also worth reminding that all policies—including those that do not include epistemic actions—have some epistemic value, because visiting any branch of the maze is informative of the choice context (because the animal collects red or white cues). Here, again, the choice is between policies that use imaginary actions to retrieve episodic content and policies that try to maximize extrinsic value directly—and which gain information only with overt actions. In situations where reward is plentiful, following a habitual policy that is insensitive to the current context is sufficient; however, despite the fact that the animal does not explicitly select epistemic or exploratory actions, its habitual behavior has nevertheless some epistemic or exploratory consequence, permitting the animal to estimate its current context at least to some extent.

This simulation highlights the fact that the choice of a deliberative strategy depends on the balance between extrinsic value (of the rewards to be collected) and epistemic value (of the possible states to be visited). In other words, in Active Inference, epistemic actions are not mandatory under residual uncertainty or when the precise value of alternatives remains to be estimated (as would be the case according to the “value-of-alternatives” theory) but they are preferred when the epistemic value is higher than the extrinsic value, because both are considered together—on a level playing field that, as shown in Equation 1, is provided by expected free energy.

With some simplifications, one can conclude that VTE behavior/forward sweeps are preferred when the cost of performing “imaginary” actions is intermediate between the values of the two branches of the maze. This is consistent with the operational definition of “costly” choices adopted in many studies of VTE (Gupta et al. 2010; van der Meer et al. 2010), where choice situations are costly if selecting the wrong option is significantly worse (i.e., in terms of delay in reward consumption) than spending time to decide. Conversely, easy choice situations are those in which one can undo or quickly recover from a wrong choice. In addition, our results suggest that the most natural way to frame interesting choice situations is in terms of relative (not absolute) utility, and more precisely as the choice between a (potential) “gain” versus a (potential) “loss,” where the cost of epistemic action is intermediate. Not only does this relative value-coding seems to apply when only one arm of the maze is baited, but also when both are baited with rewards that are significantly different—say with a smaller versus a greater reward. In this latter case, the acquisition of the smaller reward might be cast as a cost, not a gain, and hence encoded as a negative value—for example, the difference between the small reward actually acquired and the greater reward that might have been (counterfactually) secured. The fact that rodents show sophisticated forms of regret (Steiner and Redish 2014) speaks in favor of such a (counterfactually based) relative value coding. In terms of our simulations, this would imply that it is appropriate to model both “reward versus no-reward” and “high- versus low-reward” situations in terms of a gain and a loss (e.g., 2 versus −2). Rather, a value-coding that includes too many gains (e.g., 2 versus 4) might instead represent a choice situation in which reward is plentiful and exploration is not necessary (see Discussion).

More generally, the emergence of VTE in the context of differentially rewarded outcomes depends sensitively on the relative utility (i.e., prior preferences). The key thing to note here is that, in active inference, the utilities of all outcomes are relative—and utility has a quantitative meaning in terms of relative (log) probabilities of preferred states (Friston et al. 2015). In other words, because the prior preference is a probability distribution it has to sum to one, which means there is no absolute utility, only differential utilities. This is an important point, and suggests that it is the differential utility (prior preference) that determines choice behavior: this is becoming increasingly evident in neurophysiological and fMRI studies (Rushworth and Behrens 2008). In our context, the relative nature of utility spans all options—including VTE. This means that the cost of a VTE could be less than either reward, in which rewards would usually supervene. Conversely, the VTE could have a prior preference that was intermediate between the high and low reward outcomes. In this instance, one would expect qualitatively different behavior—in the sense that the cost of VTE makes it viable in relation to a potentially less preferable reward. This highlights the importance of differential utilities in determining behavior, which renders them important variables in modeling observed behavior and characterizing individuals on the basis of their choices.

#### Variants of the fourth simulation, with varying levels of extrinsic value

The fourth simulation has highlighted the fact that VTE behavior can be abolished in situations when extrinsic value overcomes epistemic value. To characterize more systematically the relationship between extrinsic value and the number of forward sweeps performed before a choice, we performed a further analysis in which we varied the amount of reward delivered by the two arms.

**Figure 7.**
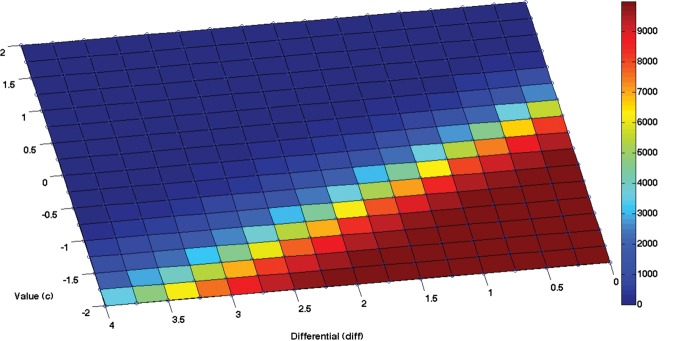
Number of policies that include forward sweeps during the fourth simulation, trial 31, as a function of the value of “usual reward” (c) and its differential with “high reward” (diff)—that is, the number *x* so that usual reward + *x* = high reward. Results are for 10,000 runs for each combination of (*c* and diff) parameters. See main text for explanation.

Specifically, we considered in more detail how the number of forward sweeps varies in trial 31 of the above simulation when we vary two parameters: the value of the “usual” reward (c) and the relative value or differential of the “high” reward (called “diff” in the figure; note that in the fourth simulation the “high” reward was twice the “usual” reward). This simulation produces a nontrivial landscape of results. As shown in Figure 7, when the “usual” reward is higher than a threshold value (here, around 1), the agent eschews imaginary actions, no matter the number of times the “high” reward exceeds it, because any policy that goes directly to any of the two arms is more valuable than all policies that include imaginary actions. However, with lower values of the “usual” reward, the agent uses imaginary actions—but only if the epistemic value (and information gain) associated with context estimation surpasses the value of the actions that lead to any reward. Specifically, the agent uses imaginary actions only if the total extrinsic value available is lower than a given value (that varies depending on c; for example, it is 2 when *c* is 0). Finally, the fact that epistemic actions are not always performed when the differential is zero shows that VTE behavior is not associated with a putative difficulty in distinguishing amongst similarly valued alternatives, as would be predicted by “value-of-alternatives” theory.

Another point that is worth noting is that the number of forward sweeps does not necessarily depend on the number of contexts to be disambiguated or (the difficulty to disambiguate) their value. Indeed, in Equation 1 epistemic value depends on the possible contexts irrespective of their specific extrinsic value. This illustrates the fact that epistemic and extrinsic value can be varied independently. Technically, expected free energy is a linear mixture of epistemic and extrinsic value, meaning that both sorts of value can be compared directly, see Equation 1. This explains the switch from deliberative to habitual behavior when the epistemic value of a deliberative behavior falls below the extrinsic value of a competing (habitual) policy. Despite so, the choice to perform or not perform epistemic actions to disambiguate context is not insensitive to extrinsic value, as ultimately in our simulations it is an increased possibility to obtain a high(er) reward that favors the selection of epistemic (or deliberative) over habitual policies. This speaks to the importance of epistemic action and exploration in the maximization of extrinsic value on a different timescale.

## Discussion

We have simulated various choice scenarios, with varying levels of extrinsic value and epistemic value afforded by uncertainty. Taken together, these simulations show that both habitual and deliberate forms of control can be accommodated under the same Active Inference scheme, where extrinsic and epistemic values are optimized together. Specifically, the balance between habitual and deliberate forms of control depends on the relative extrinsic and epistemic value that is afforded to a policy, and do not necessarily require two separate “controllers,” one for habitual choice and one for deliberate choice (the latter possibly using hippocampal sweeps) and an external arbitration mechanism as commonly assumed in reinforcement learning theories (Daw et al. 2005; Lee et al. 2014; O'Doherty et al. 2015). One problem with most “multi-controller” schemes is that they require the separate controllers to be always activated in parallel; however, in this case we would observe forward sweeps (and VTE) in all trials. One alternative view is that the controllers can be recruited serially. In this perspective, the (more demanding) deliberate processes are only recruited when necessary, for example, depending on the “value of information” (VoI) (Keramati et al. 2011; Pezzulo et al. 2013). However, to address the notoriously challenging VoI computations, one has either to assume that the deliberate system has “perfect information” about the value of choices at each state (Keramati et al. 2011) or use approximations (Pezzulo et al. 2013). In this Active Inference formulation, instead, the epistemic value of actions (which links directly to VoI computations) is automatically considered by the policy selection mechanism, which arbitrates between those that yield, or do not yield, information gain.

By the same token, in the model presented here the distinction between deliberate to habitual choice does not map one-to-one to distinct, model-based versus model-free computations. Here, both can be implemented under the same inferential scheme, by considering policies that include, or not include, epistemic actions. In other words, VTE behavior is part of a search process that—importantly—includes epistemic actions and the recall (or construction) of episodic memories. In keeping with the idea that deliberate and habitual forms of choice can recruit partially different brain networks (Balleine and Dickinson 1998; Yin and Knowlton 2006; Redish 2013), it is only in the former (deliberate) forms of choice that VTE behavior and hippocampal forward sweeps are necessary. This is coherent with previous formulations that highlight their importance in deliberative choice (van der Meer and Redish 2010; van der Meer et al. 2012; Pezzulo et al. 2014) and with empirical evidence that hippocampal processing is linked to deliberate forms of choice but not—or not necessarily—habitual forms (Redish 2013; Schmidt et al. 2013) as well as to novel information (Lisman and Otmakhova 2001; Vinogradova 2001).

Our simulations provide a normative perspective on why this is the case. The first and second simulations suggest that deliberate behavior and associated VTE and forward sweeps arise when (contextual) uncertainty has to be resolved before a choice, and it is suppressed when information gain is no longer possible (after uncertainty has been resolved). The third simulation shows that VTE behavior is key in “costly” choices, whenever a wrong choice would prevent reward consumption or delay it significantly, but is not necessarily demanded in the presence of residual uncertainty. Taken together, the results of the first three simulations link VTE behavior to behavioral flexibility, and show that while diminishing uncertainty is an important imperative in some cases (first and second simulation) it is not the primary determinant of epistemic action. Rather, it is the relative value of extrinsic versus epistemic value that regulate VTE dynamics. We further investigate this aspect in the fourth simulation, which shows that epistemic actions are suppressed when the extrinsic value that can be obtained by acting overtly (rather than covertly simulating) exceeds information gain. This latter result highlights the important role of prior preferences that, in Active Inference, are a softmax function over utilities (which determines the relative log probability of each outcome). In other words, every outcome is associated with an extrinsic value that is inherently relative to all other available outcomes. To model a preference for the greater reward, one would simply increase the differential between the two options until the lesser reward no longer predominated over epistemic value—leading to the behavior illustrated in the first simulation. Although of course there are multiple ways to set prior preferences, it is this latter value-coding, in which (with some simplifications) the value of epistemic actions is intermediate between the highest and lowest option, which we consider to be the most appropriate way to model various meaningful choice situations, including choices between a higher versus a lower reward—where, basically, the value-coding is relative, not absolute, and the low reward would be encoded as a loss compared with the other potentially (counterfactually) available reward.

The dependence of epistemic action and exploration on the overall available (extrinsic) value shows the relations between our formulation and other normative principles in foraging theory and exploration-exploitation—and especially the Marginal Value Theorem. According to the latter, an animal should stay in (exploit) its patch as long as the current reward rate—the marginal value of the patch—exceeds the average reward rate of the environment; when this is not the case, it should leave the current patch (explore) (Charnov 1976). The formal framework presented here extends these normative principles from foraging domains to VTE behavior and hippocampal forward sweeps—where the latter are assimilated to a form of “mental” exploration. In this perspective, if a habitual policy—or a policy that is insensitive to the context—provides a high reward rate, the probability of exploring should lower, unless the agent expects a change of context. This analogy remains to be fully assessed empirically.

Taken together, our results illustrate that changing environmental properties, such as the balance of extrinsic and epistemic value, demand different behavioral strategies, which—in the animal learning literature—correspond to deliberate versus habitual forms of choice (Balleine and Dickinson 1998; Dolan and Dayan 2013). In the scheme presented here, deliberative mechanisms are required when behavior needs to remain flexible and alternatives need to be explored, and it is in these conditions that VTE behavior is present. Habitual strategies emerge instead when the environment unambiguously specifies the (best) options. Finally, VTE behavior is also suppressed when the extrinsic value of any choice exceeds the information gain afforded by epistemic actions. These results provide a normative account of VTE behavior and forward sweeps that is coherent with existing evidence, and generate novel empirical predictions that can guide future research.

The theory presented here is alternative to the idea that VTE behavior stems from conditioned orienting (Bower 1959; Spence 1960) or discrimination learning (Schrier and Povar 1979). Our proposal is instead compatible with views that see hippocampal sweeps (and ventral striatal activations) as part of a model-based evaluation process (Johnson et al. 2007; van der Meer et al. 2012; Pezzulo et al. 2013; Steiner and Redish 2014), with the idea of Tolman (1948) that VTE behavior permits learning the structure of a task and flexibility theory of VTE (Regier et al. 2015). Furthermore, our proposal resonates well with the view of Johnson et al. (2012) that VTE affords “information foraging” in memory and, by engaging a model of task contingencies, permits to simulate or imagine potential observations (or information that can be sampled). These theories highlight that VTE plays several roles—in deliberation, behavioral flexibility, structure learning, information foraging—that in our proposal all stem from the fact that they afford (covert) epistemic actions. We offer a formal scheme to calculate the value of epistemic (or information foraging) actions and compare it with the extrinsic value afforded by overtly executed actions, thus selecting policies that include (deliberate) or not include (habitual) epistemic components. In this perspective, the distinguishing element of VTE behavior is the epistemic component that is not mandatory in model-based search. Furthermore, here we stress the importance of episodic components, often associated with hippocampal processing (Tulving 1972; Eichenbaum 2000), within a model-based inference scheme.

The formal model we have adopted here can, in principle, be extended in several ways. For example, we have assumed that the agent's generative model includes all the information about task contingencies, the reliability of the cues, and the number of allowable contexts, which animals must learn. Our assumptions mimic the situation faced by rodents in T-maze studies, in which the animals have already learned these probabilistic contingencies (Johnson and Redish 2007; Gupta et al. 2012). However, the computational model presented here can be easily extended to incorporate learning, as part of the Bayesian estimation scheme (Friston et al. 2015). This extension would permit to model, for example, the fact that hippocampal theta sequences, which we consider here as part of the agent's generative model, are learned through experience, albeit very rapidly (Feng et al. 2015) and before that cannot be plausibly used for episodic retrieval or simulations.

Another limitation of the current model is that we adopt a simplified method to discount the value of delayed rewards. In the model, all policies have the same length (say, three actions) and—for the sake of simplicity—we allow agents that arrive sooner to a reward site (say, after just two actions) to consume the reward more than once (say, twice, one plus the number of remaining actions in the policy). This stems from the fact that reward sites are absorbing and an agent reaching one of them cannot leave it until the end of the trial. This method permits implicitly discounting the value of a reward for the agents who arrive at the reward site later, but can be refined to obtain a better (quantitative) fit to behavioral data on temporal discounting.

Our simulations also require a notion of “context,” whose precise characterization in the brain remains to be fully spelled out. From the perspective of an animal engaged in situated action, what makes contexts different from one another is not a minor difference in perceptual features, but plausibly a meaningful difference in reward delivery—and ultimately a difference in the action to be taken. In this perspective, the view that every experimental condition (say, 3 pellets versus 1 pellet; 1 pellet versus 0 pellets) necessarily corresponds to one context for the animal appears to be an oversimplification. For example, animals could group different experimental conditions into one single context, if the strategy to be taken is essentially the same. Albeit in a different domain, the question of “when to create a new context (or task set)” has been recently posed in a series of computational studies that use Bayesian nonparametrics coupled with reward contingencies (Collins and Koechlin 2012; Collins and Frank 2013; Stoianov et al. 2015; Maisto et al. 2016). It emerges from these studies that it is often valuable to group contexts in behaviorally relevant ways, not just according to perceptual similarity of the items; for example, in such a way that a change in context necessarily signals a change in the “rule” to be applied or the strategy to be followed (Stoianov et al. 2015). In keeping, an animal might be aware that a change in the environment occurred, but might mark this event as a change of context only if it implies a change in behavioral strategy. In turn, this mechanism would require VTE and epistemic action (to infer the new context) only when there is an associated change in strategy. This hypothesis is in keeping with evidence that VTE behavior is clearly linked to changes in behavioral strategy (Regier et al. 2015), but it remains to be fully tested empirically.

Here, we have associated forward sweeps with the construction or retrieval of episodic information; for example, about past reward delivery. In the examples above, these are covert or “imaginary” actions that can be part of a deliberate strategy and a policy that the animal follows to optimize its behavior. Forward sweeps, and other forms of internally generated sequences in the hippocampus, have other roles too, besides those considered here (Diba and Buzsáki 2007; Foster and Wilson 2007; Dragoi and Tonegawa 2011; van der Meer and Redish 2010; Pfeiffer and Foster 2013; Wikenheiser and Redish 2013; Pezzulo et al. 2014). For example, trajectories can be replayed when the animal is at rest or asleep, possibly supporting a form of memory consolidation or self-training with simulated rather than real experience (Sutton 1990). The role of sleep and dreaming has been considered in terms of model optimization and complexity minimization under free energy formulations (Hobson and Friston 2012). In our model, self-training might be used to speed up learning, thus permitting more efficient reduction of residual uncertainty (e.g., about the context) and habitization. Furthermore, internally generated dynamics can support forms of spatial planning (Penny et al. 2013; Pfeiffer and Foster 2013) as well as counterfactual thinking about the value of “the path not chosen” (Steiner and Redish 2014). Incorporating all these functions in this framework is an open objective for future research.

Our model addresses only the issue of “when” (or in which conditions) VTE should be executed, not “where” it should be directed (e.g., to future locations or backward to past locations, see Steiner and Redish 2014). However, the same logic of our model can be applied to the “where” question. Learning a model of task contingencies—for example, the location where a reward is usually located and the path required to reach it—allows the animal to direct the forward sweeps to the most informative areas, or areas where the most valued information might be found. Given that the hippocampus is widely assumed to encode spatial-contextual contingencies necessary to solve navigation problems (O'keefe and Nadel 1978), this mechanism might give hippocampal sweeps their directionality; see Friston et al. (2012a) for an example of a mechanism permitting to sample from the most informative parts of the environment.

In discussing the exploration–exploitation dilemma, we made an analogy between internal and external exploration (or search) processes, the former corresponding to a cognitive form of search (here, corresponding to episodic memory or episodic future thinking, Schacter and Addis 2007; Schacter et al. 2012); and the latter corresponding to an overt exploratory behavior, as when an animal explores an unknown or less-known branch of a maze. This analogy is based on the idea that search processes in the brain and in the external environment might be formally similar (Pezzulo et al. 2013; Hills et al. 2015; Pezzulo and Cisek, 2016). First, common computational principles of information sampling might underlie both internal and external forms of exploration or search. Second, an exploration–exploitation dilemma might be intrinsic in both the decision to “stop and think” (e.g., vicarious trial and error) versus “take action immediately,” which we addressed in this article, and the decision to “explore a new option” versus “exploit the (best) known option,” which is the most traditional formulation of the dilemma. In Active Inference, these formal similarities are apparent because all forms of exploration ultimately depend on epistemic value—or more precisely, the balance between extrinsic and epistemic value—and the same mechanisms are in play when epistemic actions correspond to a memory (or prospection) process, as in the simulations presented here, or to the retrieval of cues in the external environment through overt exploration, as shown in Friston et al. (2015).

At the computational level, there are various ways to balance real (extrinsic) and epistemic actions. Some computational models (e.g., “ε-greedy”) sidestep the problem by selecting random actions with a (usually small) probability. In other models, exploration is guided by expected value; for example, in the “softmax” rule, the actions’ relative expected values determine the probability of selecting a (suboptimal) exploratory action. However, in these schemes, exploratory actions are not necessarily directed toward the most informative outcomes. Another strategy is putting a premium on actions that bring new (and useful) information; for example, assigning an “exploration bonus” to actions whose consequences are uncertain (Dayan and Sejnowski 1996) or considering in the choice the so-called “value of information” (VoI) (Howard 1966; Keramati et al. 2011; Pezzulo et al. 2013). Similarly, the scheme presented here assigns epistemic actions an explicit *value* that can be directly used to trade off policies that include or not include exploratory components (see Equation 1). In this scheme, the amount of exploration required for a given choice is itself optimized by appealing to the (normative) perspective of free energy minimization (Friston et al. 2015), which thus subsumes the calculations of information value. Furthermore, in this scheme the epistemic value of a given exploratory action can be quantified (under certain assumptions, e.g., about the prior belief of an animal) and compared against extrinsic value. This might permit designing experiments that directly test the trade-offs between exploration and exploitation, which are currently incompletely known (Daw et al. 2006; Cohen et al. 2007).

## Conclusions

How do animals balance deliberate and habitual choice strategies? Here, we have shown that this balance is mandated by different statistics of the environment and different prior beliefs entertained by the agent; in particular, by the balance of extrinsic and epistemic value. When task contingencies are uncertain or behavior needs to be flexible, epistemic actions (exploration) should be preferred; when instead the environment unambiguously specifies the best policy, behavior can be habitized (exploitation). Exploitative behavior emerges also when reward is plentiful—an idea that resonates well with classical foraging theories such as the Marginal Value Theorem. Here, we have shown context sensitive balance of deliberate and habitual choice strategies can be explained in a simple and principled way by Active Inference: namely, the maximization of expected model evidence, which comprises both extrinsic and epistemic value. We have applied this normative account to vicarious trial and error (VTE) behavior and its neurophysiological underpinnings in rodent spatial decisions.

## Materials and Methods

### Active inference

This section describes Active Inference, in which inference and behavior are seen as consequences of minimizing a variational free energy bound on surprise or, equivalently, maximizing Bayesian model evidence (Friston 2010).

*Notation*: we use conventional notation, where the parameters of categorical distributions over discrete states s∈S∈{1,…,J} are denoted by *J* × 1 vectors of expectations ⌢s∈[0,1], while the ∼ notation denotes sequences of variables over time. The entropy of a probability distribution *P*(*a*) = Pr(*A* = *a*) will be denoted by $H(A)=H[P(a)]=E_{P(a)}][-\ln P(a)]$ and the relative entropy by the Kullback–Leibler (KL) divergence $D[Q(a)||P(a)]=E_{Q(a)}[\ln Q(a)-\ln P(a)]$. The dot notation denotes *A* · *B* = *A*^*T*^*B*.

*Definition*: Active inference rests on the tuple (*P*,*Q*,*R*,*S*,*A*,*U*,Ω):

- A finite set of observations Ω
- A finite set of actions *A*
- A finite set of hidden states *S*
- A finite set of control states *U*
- A generative process over observations $\tilde{o}\in \Omega$, hidden states $\tilde{s}\in S$ and action $\tilde{a}\in A\;\;R(\tilde{o},\tilde{s},\tilde{a})=\mathrm{Pr}(\{o_{0},⇋,o_{t}\}=\tilde{o},\{s_{0},⇋,s_{t}\}=\tilde{o},\tilde{s},\{a_{0},⇋,a_{t-1}\}=\tilde{a})$.
- A generative model over observations $\tilde{o}\in \Omega$, hidden $\tilde{s}\in S$ and control $\tilde{u}\in U$ states $P(\tilde{o},\tilde{s},\tilde{u}|m)=\mathrm{Pr}(\{o_{0},\ldots ,o_{T}\}=\tilde{o},\{s_{0},\ldots ,P(s_{T}\}=\tilde{s},\{u_{t},\ldots ,u_{T}\}=\tilde{u})$, with parameters θ.
- An approximate posterior over hidden and control states such that $Q(\tilde{s},\tilde{u})=\mathrm{Pr}(\{s_{0},⇋,s_{T}\}=\tilde{s},\{u_{t},⇋,u_{T}\}=\tilde{u})$ with parameters or expectations (⌢s,⌢π), where π∈{1,…,K} is a policy that indexes a sequence of control states $\left(\tilde{u}|\pi )=(u_{t},⇋,u_{T}|\pi \right)$.

*Remarks*: the generative process describes transitions among hidden states that depend upon action, where hidden states generate outcomes that the agent observes. Actions are sampled from approximate posterior beliefs about control states based on the agent's generative model (denoted by *m*) of the generative process. Approximate posterior beliefs about states are encoded by expectations. Note that in this formulation there is a distinction between actions (that are part of a generative process) and control states (that are part of the generative model). This distinction allows actions to be sampled from posterior beliefs about control, effectively converting an optimal control problem into an optimal inference problem (Attias 2003; Botvinick and Toussaint 2012; Kappen et al. 2012; Solway and Botvinick 2012; Donnarumma et al. 2016). Furthermore, note that (unlike the generative process) the generative model includes beliefs about future states.

To couple the agent to its environment, one has to specify how its expectations depend upon observations and how its action depends upon expectations. In discrete formulations, expectations minimize variational free energy and the ensuing expectations of control states prescribe action at the current time *t*:

$$\begin{array}{r} \left(⌢\;s{\;}^{*},⌢\;\pi {\;}^{*})=\arg\min F(\tilde{o},⌢\;s,⌢\;\pi \right)\\ \mathrm{Pr}(a_{t}=u_{t})=Q(u_{t}|{⌢\;\pi }^{*})\\ F(\tilde{o},⌢\;s,⌢\;\pi )=E_{Q}[-\ln P(\tilde{o},\tilde{s},\tilde{u}|m)]-H[Q(\tilde{s},\tilde{u})]\\ =-\ln P(\tilde{o}|m)+D[Q(\tilde{s},\tilde{u})||P(\tilde{s},\tilde{u}|\tilde{o})]. \end{array}\;\left(2\right)$$

In brief, at each choice point, the agent figures out which states are most likely by optimizing its expectations with respect to variational free energy (using the generative model). After optimizing its posterior beliefs, an action is sampled from the posterior marginal over control states. Given this action, the environment generates a new observation (using the generative process) and a new cycle starts.

The first expression for free energy in Equation 2 shows that it is an expected energy, under the generative model, minus the entropy of the approximate posterior. This expression can be rearranged to give the second expression, which shows that free energy is an upper bound on the negative logarithm of Bayesian model evidence $-\ln P(\tilde{o}|m)$, also known as surprisal (or surprise). This is because the divergence term cannot be less than zero (Beal 2003). Therefore, minimizing free energy corresponds to minimizing the divergence between the approximate and true posterior. This formalizes the notion of approximate Bayesian inference in psychology and machine learning (Helmholtz 1866; Dayan et al. 1995; Dayan and Hinton 1997).

However, here the objective is not simply to infer hidden states but to actively sample, by selecting an appropriate sequence of actions, outcomes that minimize free energy—or in other words, for an agent, to take action that leads to a small number of preferred outcomes (goals). In Active Inference, this corresponds to the fact that agents minimize surprisal, while a priori expecting to sample preferred outcomes (goals).

The generative model includes the agent's prior belief, which can be decomposed into: a likelihood over outcomes, a prior over state transitions and the prior over control states. These correspond to the three marginal distributions of the generative model: $P(\tilde{o},\tilde{s},\tilde{u}|m)=P(\tilde{o}|\tilde{s})P(\tilde{s}|\tilde{u})P(\tilde{u}|m)$. Crucially, the only self-consistent prior belief an agent can entertain is that control states will minimize free energy, i.e., that it will achieve its goals. One can express this formally by assuming a Boltzmann form for the prior over control states based on the path integral of expected free energy from the current to the final state (cf., Hamilton's principle of least action). As sequences of control states correspond to policies, we will express this in terms of the quality Q (aka value or negative free energy) of a policy

$$\begin{array}{rl} & Q(\tilde{u}|\pi ):=Q(\pi ):\\ \ln P(\tilde{u}|\gamma )=\gamma \cdot & Q(\pi )=\gamma \cdot (Q_{t+1}(\pi )+⇋+Q_{T}(\pi ))\\ Q_{\tau }(\pi ) & =E_{Q(o_{\tau },s_{\tau }|\pi )}[\ln P(o_{\tau },s_{\tau }|\pi )]+H[Q(s_{\tau }|\pi )]. \end{array}\;\left(3\right)$$

Heuristically, this means that agents believe they will pursue policies that lead to their goals (or more formally, minimize expected free energy in the long run and—because “unsurprising” outcomes correspond to the agent's goals—implicitly minimize their surprise about those outcomes). Note that the expected free energy is the free energy of current beliefs about hidden states in the future (i.e., the beliefs that an agent entertains while planning), not the free energy of future beliefs. More formally, the expected free energy (negative value) is the energy of counterfactual outcomes and their causes expected under their posterior predictive distribution minus the entropy of the posterior predictive distribution over hidden states. The posterior predictive distributions are distributions over future states expected under the (approximate) posterior distribution: $Q(o_{\tau },s_{\tau }|\pi )=E_{Q(s_{t})}[P(o_{\tau },s_{\tau }|s_{t},\pi )]$. In this setup, γ ∈ θ plays the role of a sensitivity or inverse temperature parameter that corresponds to the precision of, or confidence, in prior beliefs about behavior, and influences action selection (the Softmax rule) so that policy selection becomes more deterministic with increased confidence.

Rearranging the expression for expected value above reveals two intuitive terms; namely, extrinsic and epistemic value.

$$\begin{array}{r} Q_{\tau }(\pi )=E_{Q(o_{\tau },s_{\tau }|\pi )}[\ln P(o_{\tau },s_{\tau }|\pi )-\ln Q(s_{\tau }|\pi )]\\ =E_{Q(o_{\tau },s_{\tau }|\pi )}[\ln Q(s_{\tau }|o_{\tau },\pi )+\ln P(o_{\tau }|m)-\ln Q(s_{\tau }|\pi )]\\ =\underset{\mathrm{Extrinsic}\;\mathrm{value}}{\underbrace{E_{Q(o_{\tau }|\pi )}[\ln P(o_{\tau }|m)]}}+\underset{\mathrm{Epistemic}\;\mathrm{value}}{\underbrace{E_{Q(o_{\tau }|\pi )}[D[Q(s_{\tau }|o_{\tau },\pi )||Q(s_{\tau }|\pi )]]}}. \end{array}\;\left(4\right)$$

Here, the generative model of future states $P(o_{\tau },s_{\tau }|\pi =Q(s_{\tau }|o_{\tau },\pi ))$ $\left(o_{\tau }|m\right)$ comprises the predictive posterior and prior beliefs about counterfactual outcomes that determine extrinsic value. Extrinsic value is the negative cost or utility $C(o_{\tau }|m)=\ln P(o_{\tau }|m)$ of an outcome expected under its posterior predictive distribution. This generative model means that the agent considers costly outcomes surprising (in the technical sense of being not “compatible” with prior beliefs, which—essentially—encode goals), irrespective of the policy in question (that is, the agent believes all unsurprising policies will lead to the same preferred outcomes that represent the agent's goals).

Under this (predictive) generative model, the quality of a policy can be expressed in terms of extrinsic and epistemic value:

Extrinsic value: this is the expected utility of future outcomes (c.f., reward) expected under the posterior predictive distribution. This means that agents (believe they) will maximize expected utility to ensure preferred (low cost) outcomes. Technically, the preferred outcomes are expressed as a probability distribution over the agent's observations (not states). Of note, these probabilities are normalized—so that it is their relative and not their absolute value that is most meaningful.

The minimization of the distance between expected and preferred outcomes can be seen more clearly when hidden states are observed unambiguously. In this special case, *S*_τ_ = *O*_τ_ and the value of a policy reduces to the (negative) Kullback–Leibler divergence:

$$\begin{array}{rl} Q_{\tau }(\pi ) & =\underset{\mathrm{Extrinsic}\;\mathrm{value}}{\underbrace{E_{Q(o_{\tau }|\pi )}[\ln P(o_{\tau }|m)]}}+\underset{\mathrm{Epistemic}\;\mathrm{value}}{\underbrace{H[Q(o_{\tau }|\pi )]}}\\ & =-\underset{\mathrm{KL}\;\mathrm{ddivergence}}{\underbrace{D_{KL}[Q(o_{\tau }|\pi )||P(o_{\tau }|m)]}}. \end{array}\;\left(5\right)$$

Epistemic value: epistemic value is the expected information gain under predicted outcomes. In other words, it reports the reduction in uncertainty about hidden states afforded by observations. Because the KL divergence (or information gain) cannot be less than zero, the minimum information gain obtains when the posterior predictive distribution is not informed by a new observation. Heuristically, this means valuable policies will search out observations, cues or “signs” that resolve uncertainty about the state of the world (e.g., in this context, retrieving from memory past episodes or simulating them, in order to resolve uncertainty about the hidden location of food).

As discussed earlier, expected free energy is a linear mixture of epistemic and extrinsic value. This implies that, as illustrated by our simulations, the balance between extrinsic value (of the rewards to be collected) and epistemic value (of the possible states to be visited) is context dependent. When accurate state estimation is required to secure a reward, it is likely that epistemic value dominates policy selection. On the contrary, extrinsic value will likely dominate policy selection in at least two occasions. First, when there is no posterior uncertainty and the agent is confident about the state of the world, because essentially there can be no further information gain and epistemic value will be the same for all policies. In this case, exploration is not necessary and behavior can be habitized. Second, when reward is plentiful and the agent can select high-value policies without disambiguating its state—hence, even in this case, exploratory actions are superfluous and policies including them will not be selected. In sum, in this model, it is the relative weight of epistemic and extrinsic value that promotes or prevents exploration, in both its overt and covert (VTE) forms.

## Footnotes

5 Formally, this process corresponds to the minimization of (expected) free energy, i.e., a mathematical quantity, not to be confused with an animal's metabolic resources, see Friston (2010).

6 Formally, the probabilistic distance between the actually sensed outcomes and the (rewarded) outcomes expected under the prior probability can be encoded as a “surprisal” (or surprise); and for this, the minimization of surprisal is valuable (i.e., zero surprisal means that the animal is in the goal state). Note that this is a technical use of the notion of surprisal and it does not always correspond to the commonsense use of “surprise.”
